# Tumor‐associated exosomes in cancer progression and therapeutic targets

**DOI:** 10.1002/mco2.709

**Published:** 2024-09-07

**Authors:** Xiaomin Liu, Fan Wu, Wei Pan, Guangchao Liu, Hui Zhang, Dawei Yan, Saijing Zheng, Zhongliang Ma, Xiaojun Ren

**Affiliations:** ^1^ Lab for Noncoding RNA & Cancer School of Life Sciences Shanghai University Shanghai China; ^2^ Shanghai New Tobacco Product Research Institute Co., Ltd. Shanghai China; ^3^ Department of Chemistry College of Chemistry and Life Sciences Beijing University of Technology Beijing China

**Keywords:** cancer progression, regulation, therapeutic targets, tumor‐associated exosomes

## Abstract

Exosomes are small membrane vesicles that are released by cells into the extracellular environment. Tumor‐associated exosomes (TAEs) are extracellular vesicles that play a significant role in cancer progression by mediating intercellular communication and contributing to various hallmarks of cancer. These vesicles carry a cargo of proteins, lipids, nucleic acids, and other biomolecules that can be transferred to recipient cells, modifying their behavior and promoting tumor growth, angiogenesis, immune modulation, and drug resistance. Several potential therapeutic targets within the TAEs cargo have been identified, including oncogenic proteins, miRNAs, tumor‐associated antigens, immune checkpoint proteins, drug resistance proteins, and tissue factor. In this review, we will systematically summarize the biogenesis, composition, and function of TAEs in cancer progression and highlight potential therapeutic targets. Considering the complexity of exosome‐mediated signaling and the pleiotropic effects of exosome cargoes has challenge in developing effective therapeutic strategies. Further research is needed to fully understand the role of TAEs in cancer and to develop effective therapies that target them. In particular, the development of strategies to block TAEs release, target TAEs cargo, inhibit TAEs uptake, and modulate TAEs content could provide novel approaches to cancer treatment.

## Graphical abstract

1. Generalized the biogenesis, composition, and function of tumor‐associated exosomes (TAEs) in cancer progression.

2. Highlight potential therapeutic targets of TAEs.

3. TAEs are emerging as important mediators of cancer progression and potential therapeutic targets.

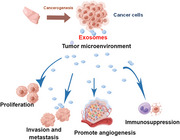

## 1 INTRODUCTION

With the gradual improvement of detection methods, targeted therapies, and immunotherapy, the mortality rate of cancer patients has decreased somewhat.^1^, ^2^ Nevertheless, cancer continues to be the leading cause of death for both males and females in US statistics.^3^ The American Cancer Society's recently released “Blueprint for Cancer Prevention and Mortality Reduction 2030” emphasizes three key elements of cancer mortality reduction: prevention, screening, and treatment.^4^ Targeted therapy is an important means to achieve targeted early treatment of malignant tumors, but there are fewer mature therapies targeted that can be used to guide clinical practice. Several methods have been proposed for early diagnosis and treatment of cancer,^5^ but their clinical opportunity is still unknown due to the lack of standardized tests and high testing costs.^6^, ^7^ Therefore, the search for new stable and reliable targeted therapies for tumors remains an urgent problem in oncology research, which will aid in its diagnosis and treatment.

Exosomes are cell‐secreted nanoparticles (approximately 40–160 nm) that are involved in several functions such as intercellular communications, biological processes, and cell signaling.^8^, ^9^ The biogenesis and functionality of exosomes have been described, such as containing a variety of biologically active molecules to function, removing excess or unnecessary components from cells to maintain cell homeostasis, and serving as tools for intercellular material and information exchange.^10^, ^11^ A variety of functional biomolecules in donor cells are delivered and internalized by exosomes to perform functions such as gene regulation and modification of protein factors in recipient cells. Cancer‐associated factors in lung cancer have been shown to be associated with onset, metastasis, treatment, and prognosis through the transfer and recruitment of lung cancer‐derived exosomes to other cells.^12^, ^13^ Due to the surface alterability and good biocompatibility of endogenous exosomes, they are widely used as vehicles for drug delivery compared with liposomes.^14^ Studies have shown that extracellular vesicles (EVs) containing the CRISPR/Cas9 gene editing system were targeted by aptamers recruitment to the lesion for precise gene editing for cancer treatment.^15^ Thus, in the future, exosomes could serve as vectors for drug delivery, offering a potential treatment choice for cancer.

Exosome is a nanoscale bilayer lipid inclusions structure that can carry characteristic biological information molecules of the parent cell, such as proteins, lipids, deoxyribonucleic acid (DNA), mRNA, noncoding RNAs (ncRNAs), and so on, which are widely existed in body fluids, including blood, tears, urine, saliva, breast milk, and ascitic fluid. Of these, ncRNAs, including microRNAs (miRNAs), circular RNAs (circRNAs), long noncoding RNAs (lncRNAs), tRNA‐derived RNA fragments (tRFs), and other common RNA fragments with some other specific functions, have complex interactions with each other.^16^ They are mainly centered on miRNAs, forming a network of interactions that target genes and profoundly affect transcriptional and expression activities.^17^ The progression of cancer involves multiple interactions of ncRNAs, which key nodes of the ncRNAs network may play a role in promoting or suppressing cancer.^18^ Therefore, ncRNAs are considered to be important biomarkers and potential treatment targets for cancer.^19^ Tumor‐derived exosomes contain ncRNAs with highly reactive activity to cancer‐promoting stress conditions such as hypoxia,^20^ and they are regarded to be crucial elements in regulating lung carcinogenesis in cellular communication.^21^, ^22^ Due to the deliverability and modifiability of exosomes, they are suitable for carrying oncogenic ncRNAs to target cancer cells, and the bilayer membrane of exosomes ensures that ncRNAs are not degraded.^23^ In current clinical diagnosis and therapy, tumor‐derived exosomes‐ncRNAs have potential significance.^24^

In this review, we summarize the characteristics of exosomes, the role of tumor‐associated exosomes (TAEs) and nucleic acid (ncRNAs) in cancer progression, and emphasize the therapeutic targeting of TAEs. With the continuous progress of science and technology and the depth of clinical trials, we have reason to believe that exosomes will become one of the important tools for cancer treatment in the future.

## 2 CHARACTERISTICS AND IDENTIFICATION OF EXOSOMES

Exosomes, approximately 40–160 nm in size, are a class of EVs wrapped in a phospholipid bilayer and secreted by almost all eukaryotic cells that encapsulate abundant active biomolecules and compounds.^25^ Exosomes are very heterogeneous due to their biogenesis involving heterogeneous membrane invaginations, differences in surface functional proteins and inclusions, and the influence of the different cellular microenvironments in which they are secreted.^8^ In addition, the different internalization mechanisms of recipient cells allow exosomes to exhibit more complex biological functions.

### 2.1 Mechanisms of exosome formation in tumor cells

The mechanisms of exosome genesis include the formation of intracellular bodies, the formation of multivesicular bodies (MVBs) and secretion. Intraluminal vesicles (ILVs) formed in MVBs are the precursors of exosomes. Cytoplasmic membrane undergoes invagination and wraps around the cell membrane surface binding proteins with some components of the extracellular matrix. This process forms early sorting endosomes. RNA derived from the nucleus with ribonucleoprotein (RNP), golgi‐sorted protein, and lipid components enter the late sorting endosomes after maturation, forming MVBs that house multiple ILVs containing multiple bioactive molecules.^26^, ^27^ The formation of ILVs and MVBs was intricately regulated by signaling molecules, containing classical pathway dependent on endosome sorting complex required for transport (ESCRT)^28^ versus the ESCRT‐independent lipid valve‐induced pathway.^29^ Subsequently, these MVBs either fuse with lysosomes, leading to degradation, or with the plasma membrane, leading to the release of their internal vesicles, that is, exosomes.^30^

### 2.2 Composition of exosome cargo and its functional roles in cancer progression

More and more studies have shown that exosomes are involved in malignant phenotypes of cancer, such as proliferation, metastasis, and drug resistance, in addition to epithelial–mesenchymal transition (EMT), angiogenesis, and other processes. Exosomes are widely distributed and studies have detected exosomes from tumor tissues and cells. It has also been found that exosomes can exchange substances between tumor cells or tumor microenvironment (TME), thus exerting a cancer‐promoting or cancer‐suppressing effect. Exosomes released by tumor cells, such as miRNAs, circRNA, and lncRNAs, are important mediators of cell–cell communication in the TME and have recently been demonstrated to play an important role in the regulation and communication of tumor development and progression.^8^ Here, we will briefly describe the role of ncRNAs function, and we will elaborate later.

Studies have indicated that miR‐21 carried in exosomes produced by hepatocellular carcinoma (HCC) cells could convert hepatic stellate cells into tumor‐associated fibroblasts, thereby promoting cancer progression.^31^ HCC cells could evade tumor immune surveillance via exosomes and could also promote growth and survival via exosomal‐cyclic RNA produced by adipocytes.^32^, ^33^ Cheng et al.^34^ showed that HCC cell‐derived exosomes can act on macrophages to reduce the secretion of IL‐6, IL‐1β, IL‐10, and TNF‐α, thereby activating the signal transduction and activator of transcription 3 (STAT3) pathway and upregulating PD‐L1 expression. HCC cell‐derived exosomes containing miR‐92b could inhibit CD69 expression, counteract the cytotoxic effects of NK cells, and reduce T lymphocyte activation.^34^, ^35^ Zhu et al.^36^ demonstrated that exosome miR‐106a was able to induce MMT to promote peritoneal metastasis of gastric cancer (GC) by activating the TGF‐β pathway through targeting Smad7 and tissue inhibitory factor of metalloproteinases 2. Study demonstrated that nicotinamide N‐methyltransferase‐containing exosomes from GC cells promoted peritoneal metastasis through TGF‐β/Smad2 signaling.^37^ Kimura et al.^38^ found that exosome miR‐29b from mesenchymal stem cells (MSCs) was able to inhibit peritoneal metastasis of GC by inhibiting MMT by blocking TGF‐β1 signaling. Qiu et al.^39^ verified that exosome miR‐519a‐3p promoted GC liver metastasis by activating the dual‐specificity phosphatase 2‐mitogen‐activated protein kinase (MAPK)/extracellular signal‐regulated kinase (ERK) axis in intrahepatic macrophages. Yang et al. found that the exosome‐circRNA ubiquitin splicing enzyme E2Q2 (circ UBE2Q2) could inhibit autophagy and promote glycolysis, activate STAT3 signaling pathway and EMT, and promote GC liver metastasis.^40^ Zhang et al. verified that exosome circFCHO2 activated JANUS kinase 194 (JAK194)/STAT5 pathway by sponging miR‐1‐3p and promoted GC lung metastasis.^41^ Study also reported that lncRNAs are involved in tumor angiogenesis, which is an important link in maintaining tumorigenesis and promoting tumor metastasis.^42^ The exosome‐derived lncRNA FAM225A further enhanced the expression of NETO2 and FOXP1 by regulating miR‐206, thereby promoting esophageal squamous cell carcinoma progression and angiogenesis. It has been shown that exosome‐derived lncRNA RPPH1 can act on β‐III microtubule proteins and exert an inhibitory effect on their ubiquitination, which can induce the production of EMT in colorectal cancer (CRC) cells, and consequently, participate in the process of CRC progression and metastasis in vivo.^43^ The exosome circPABPC1 induces EMT production and promotes CRC liver metastasis by promoting the expression of HMGA2 in the nucleus as well as playing a role in the cytoplasm of BMP4 and ADAM19.^44^ Fibroblasts are the most abundant stromal cell population in a variety of tumors and are closely related to tumorigenesis and progression, whereas cancer‐associated fibroblasts (CAFs) are formed by the transformation of normal fibroblasts induced by tumor cell‐derived exosomes. Compared with normal cells, the exosomes of CAFs contain different molecules, such as growth factors and miRNAs, which have different effects on target cells in the TME and can stimulate tumor growth and metastasis.^45^ Tang et al.^46^ revealed that miR‐208a could affect the proliferation and radiosensitization of human lung cancer cells through exosomal translocation, which in turn targets p21. Fan et al.^47^ found that the proangiogenic effect of exosome miR‐210, a source of lung cancer cells, on CAFs may be through the modulation of the JAK2/STAT3 signaling pathway and TET2 in CAFs. You et al.^48^ demonstrated that exosomes derived from CAFs were associated with EMT in lung cancer cells, and their study found that CAFs were able to exosomally deliver SNAI1 molecular mechanism of delivering SNAI1 to recipient cancer cells, that is, lung cancer cells, which in turn induces EMT in cancer cells. Qi et al.^49^ elucidated that plasma exosome miR‐660‐5p promotes tumor growth and metastasis in NSCLC. Macrophage‐derived exosomes stimulated by LPS were able to activate the TGF‐β/Smad2/3 signaling pathway, which increased the expression of EMT‐related proteins vimentin, sma, and Col1 in A549 cells.^50^

### 2.3 Specific markers of tumor‐derived exosomes

Over the last two decades, several tetratransmembrane proteins, in particular CD63, CD81, and CD9, have been used as markers of exocytosis because of their accumulation in small EVs compared with whole‐cell lysates, as well as the steady‐state accumulation of CD63 in MVBs. However, their presence in other EVs has recently been observed. By capturing EVs specifically carrying CD63 or CD9 or CD81 and then analyzing their protein composition and enrichment for endosomal markers, it was proposed that EVs containing only CD9 or CD81 without CD63 may not form in endosomes, whereas those carrying CD63 along with one or two other tetraspanning proteins may correspond to endosome‐derived ectosomes.^51^

In recent years, many studies have shown that exosomes play an important role in tumorigenesis, progression, and response to treatment and have been considered as potential predictive markers of treatment efficacy.^52^ For example, after radiotherapy, tumor cells may release a number of exosomes associated with DNA damage and these exosomes can be used to assess how well a tumor responds to radiotherapy. In particular, exosomes contain a variety of tumor‐related protein markers, such as human epidermal growth factor receptor 2(HER2), EGFR, glypicans, integrin, and programmed death ligand 1 (PD‐L1). These markers are overexpressed or abnormally secreted in tumor cells, so detecting their levels in exosomes can help doctors diagnose the type of tumor and degree of progression more accurately. Not only that, these exosomal protein markers can also be used to predict tumor treatment response. The most typical study is that the dynamic change of plasma exosome PD‐L1 before and after anti‐PD‐1 treatment can be used as a predictive marker to monitor patients’ treatment response.^53^, ^54^

The tumor susceptibility gene 101 (TSG101) and the associated proteins ALG2‐interacting protein X are specific markers for exosome identification.^55^ Some cells secrete exosomes without relying on any fine molecular regulation, which was a reflection of exosome heterogeneity.^56^ After the formation of MVBs, degradative MVBs were translocated to lysosomes to be degraded and may be dependent on the binding of autophagosomes present in the cell. Degradation products are recycled by the cells. Based on the transport of the microtubule network and cytoskeleton, secretory MVBs are transported to and integrated with the phospholipid bilayer on the cell surface, where ILVs are released to form exosomes with lipid bilayers.^57^

## 3 THE ROLE OF TAEs IN CANCER PROGRESSION

Among tumor progression, exosomes play a critical role in information exchange, serving as the medium of intercellular communication.^58^ The progression of cancer cells is not random. Cancer cells will be filtrated by relevant molecular programs to select specific target organs and metastasized to appropriate target organs. Exosomes also act as a crucial role in physiological and pathological process.^59^, ^60^ On the one hand, the physiological response require maintenance of exosome secretion,^61^ and the pathological state is characterized by exosomes‐induced carcinogenesis, proliferation, immunosuppression, migration, invasion, and angiogenesis.^62^, ^63^

### 3.1 Effects of exosome‐mediated communication on tumor growth and metastasis

TAEs, as important mediators of information transfer from tumor cells, regulate biomolecular interactions and gene expression through autocrine or paracrine secretion in tumor growth and migration, affecting tumor biological phenotypes, including the induction of inflammation, angiogenesis, and EMT, and ultimately regulating tumor growth and metastasis.^64^, ^65^ The process of tumor development requires the involvement of a variety of biological substances and TAEs participate in gene regulation by carrying them. Therefore, body fluids from cancer patients exhibit a higher concentration of exosomes compared with those from healthy indivisuals.^66^ In preclinical studies, Liang et al.^67^ indicated that inflammasome signaling is activated by tumor‐derived exosomal TRIM59, which regulates ABHD5 proteasomal degradation to promote tumor growth and accelerates cancer progression by secreting IL‐1β. In other words, these data studies indicated that exosomes play a significant role in cancer progression.^68^

Dysregulation of signaling pathways mediates an overall change in the pattern of molecular regulatory networks, an important molecular biological mechanism mediated by multiple factors, and induces tumor growth and metastasis.^69^, ^70^ More and more evidence suggest that the growth of tumors is accelerated by excessive release of exosomes from tumor cells.^65^ Research shown a CRC‐derived exosome containing human antigen R promotes lung cell proliferation by increasing the stability of mRNA for the cell cycle signaling pathway‐related factors c‐Myc and downregulating the expression of the cycle surveillance factor p21, altering the biological process.^71^ In the progression of nasopharyngeal carcinomas (NPCs), tumor cells activate the Akt/ERK signaling pathway through the secretion of TAEs delivering Epstein–Barr virus‐encoded latent membrane protein 1, which promotes proliferative migration and neovascularization in NPCs.^72^ Upregulation of Wnt5b is associated with cancer invasiveness. Chinese hamster ovary cells are activated by exosomes from A549 lung adenocarcinoma (LUAD) cells and their migration and proliferation is stimulated by exosomes derived from human pancreatic cancer (PC) cells, PANC‐1. Caco‐2/Wnt5b cells were induced to migrate and proliferate using Caco‐2 colon cancer cells, while A549 cells were stimulated by Wnt5b exosomes.^73^ As a result of these findings, it can conclude that Wnt5b‐associated exosomes may be paracrine in their effect on cell migration and proliferation. Additionally, Huang et al.^74^ found that M2‐like TAMs promote the migration and invasion of cancer cells through intercellular delivery of M2‐like macrophage‐derived exosomes (M2‐exos) in vitro and in vivo. They demonstrated that the effect of M2‐like macrophage‐mediated invasion and migration on NSCLC cells was significantly reduced after blocking ITG αVβ3. This study reveals blocking exosomal ITG αVβ3 could be a potential treatment option for preventing tumor metastasis.

### 3.2 Influence of tumor‐derived exosomes on the TME

**FIGURE 1:**
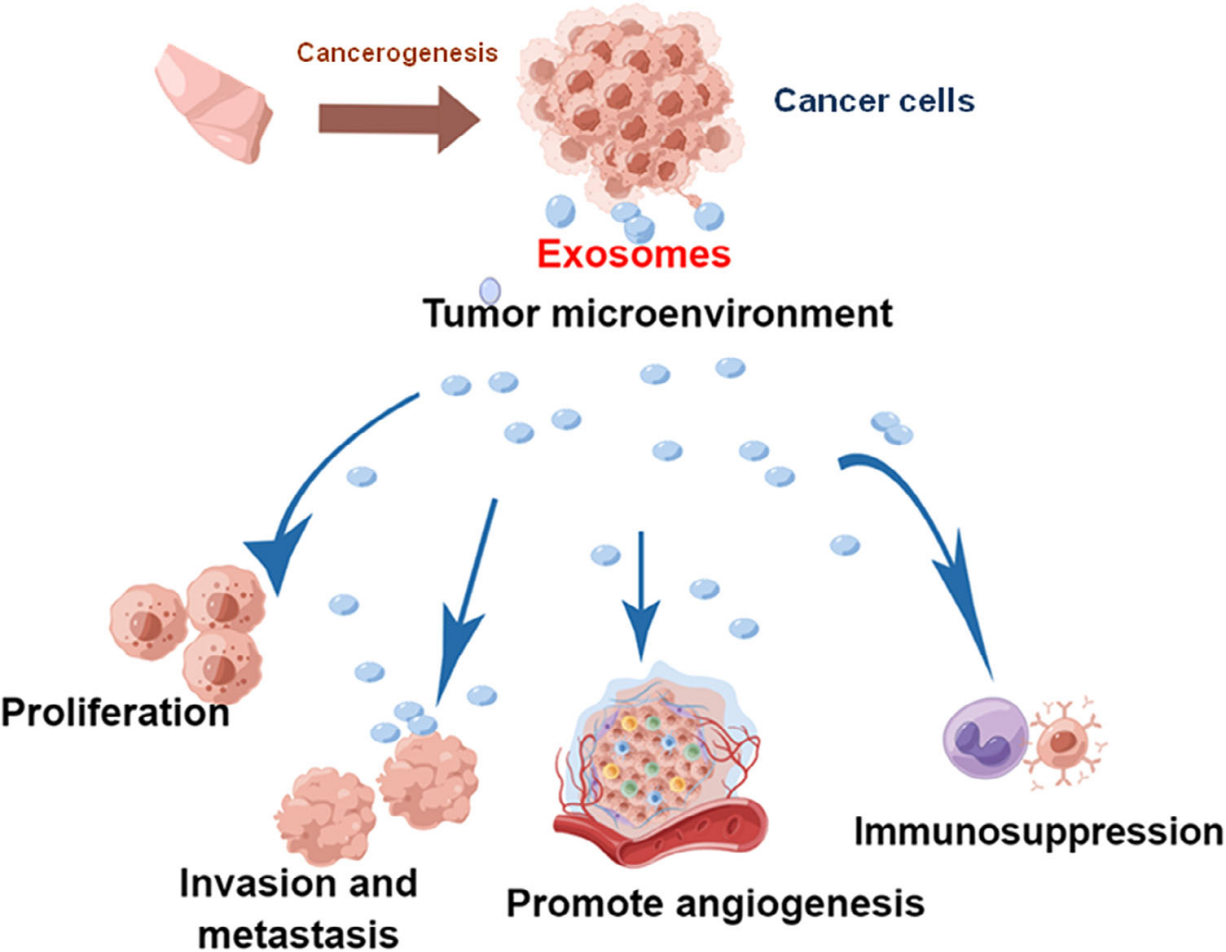
Tumor exosomal regulates cancer progression, like proliferation, invasion, metastasis, angiogenesis, and immunosuppression.

TAEs promote the occurrence and development of cancer by increasing the formation of the cancer microenvironment, promoting the ability of tumor cell invasion and metastasis, and mediate tumor immuno‐suppression.^75^ Cancer microenvironment consists of extracellular matrix, fibroblasts, and perivascular cells, especially highly active immune cells.^76^ Studies have shown that exosomes from irradiated lung cancer cells regulated the migration of recipient cells by accelerating glycolytic process.^77^ Some studies suggest that exosomes act as an important communication media between different cell types in the TME.^78^, ^79^ Exosomes, as a novel intercellular communication mechanism, can transfer bioactive molecules from one cell to another, resulting in the exchange of genetic information and reprogramming of recipient cells.^80^ In addition, there is growing evidence that macrophages are the most abundant group of white blood cells in lung cancer and play a key role in every stage of cancer progression.^81^ These tumor‐associated macrophages (TAMs) promote tumor transformation, tumor immune escape, and subsequent metastasis cascade. It follows that tumor‐associated exosomes can regulate tumor progression, such as invasion, metastasis, angiogenesis and immunosuppression, and thus regulate tumor development (Figure 1).

During tumor growth and migration, tumors undergo inflammatory (M1) and regulatory (M2) polarization by reprogramming the metabolism of TAMs, modulating gene expression, and altering immune properties.^82^ They act as a crucial regulatory role in the TME for tumor development. M1 polarization of TAM mediates the inflammatory effects of tumors through the secretion of inflammatory factors such as TNF‐α and IL‐6 and exhibits a biological function to inhibit cancer. However, at the end stage of tumor development, the M1 polarization of TAM is often reprogrammed by tumor cells toward M2 characteristics, most notably through changes in membrane markers and secreted factors. M2 polarization is characterized by inhibition of inflammation, promotion of tumor growth and migration, and induction of drug resistance. Although TAMs exhibit an intermediate state between M1 and M2 in some specific states, M2 polarization is often the final destination of TAMs in tumor development.^83^, ^84^ Recent studies have demonstrated the existence of complex cellular communication mechanisms in tumor‐mediated polarization of TAMs. TAEs, as important cellular communication mediators, mediate the exchange of information between tumor cells and TAMs through the delivery of biomolecules, which in turn reprograms the metabolism and regulates gene expression of TAMs.^85^, ^86^ Therefore, the regulation of TAMs polarization at the stage of tumor development is crucial and TAE‐mediated cellular communication plays an important role.

As mentioned earlier, ncRNAs are extremely important information carriers in TAEs. In the TME, TAMs undergo changes in their biological properties in response to tumor development, and tumor‐derived ncRNAs are delivered via TAEs and mediate TAMs polarization. Among them, miRNAs are the most studied class and are delivered to TAMs to mediate gene silencing functions. Among gynecological tumors, triple‐negative breast cancer (TNBC) and ovarian cancer (OV) are the most serious types of cancer. miR‐184‐3p enters TAMs via tumor‐derived exosomes and targets EGR1 to inhibit the c‐Jun N‐terminal kinase (JNK) signaling pathway, inducing M2 polarization in TAMs and promoting TNBC growth and metastasis.^87^ In the hypoxic TME, miR‐1225‐5p was enriched in tumor‐derived exosomes and downregulated the expression of Toll‐like receptor 2 expression (TLR2) to promote M2 polarization of TAMs, which promoted the development of OV through activation of the WNT signaling pathway.^88^ Exosomes in the microenvironment of nervous system‐associated tumors undergo complex informational crosstalk with different neuronal cells and are significantly associated with the polarization of TAMs. For example, let‐7i‐5p and miR‐221‐3p induces M2 polarization and promotes medulloblastoma development by downregulating peroxisome proliferator activated receptor gamma (PPARγ) after delivery to TAMs via exosomes.^89^ Among neurological tumors, the deadliest are gliomas. miR‐3591‐3p was significantly enriched in TAEs and targeted and downregulated the CBLB of TAMs, activating the JAK2/STAT3 and MAPK signaling pathways to cause M2 polarization and promote glioma progression.^90^ TAMs are important players involved in the gradual regulation of the TME in the development of CRC, which are usually regulated by TAEs. miR‐934 is highly expressed in CRC‐derived exosomes and is translocated to TAMs to silence PTEN as well as activate the phosphatidylinositol 3‐kinase (PI3K)/AKT signaling pathway to promote M2 polarization. Meanwhile, TAMs induced CRC to produce miR‐934‐rich exosomes by secreting CXCL13 positively counteracting the regulatory effects of CRC.^91^ They promote the EMT process and migration of CRC by participating in the regulation of the TME. The TME is particularly important for the regulation of lung cancer development. For example, LUAD translocates miR‐19b‐3p via exosomes, downregulates PTPRD in TAMs and mediates activation of STAT3 dephosphorylation, leading to M2 polarization. LUAD inhibits its proteasomal degradation through exosomal transport of miR‐19b‐3p, which downregulates PTPRD and mediates STAT3 dephosphorylation activation in TAMs,^92^ as well as transport of miR‐3153, which downregulates zinc finger protein 91 in TAMs, resulting in deubiquitination of misshapen‐like kinase 1, inhibiting its proteasomal degradation pathway, activating the JNK signaling pathway,^93^ ultimately leading to M2 polarization and promotes the development of LUAD. The mechanisms by which lncRNAs regulate TAMs via TAEs are mainly sponge adsorption of miRNAs and binding to critical protein factors. LINC00313 sponges miR‐135a‐3p through TAE transport and upregulates STAT6, inducing M2 polarization of TAMs and promoting NSCLC progression.^94^ FGD5‐AS1 is enriched in PC‐derived exosomes and interacts with p300 of TAMs to activate the STAT3/NF‐κB pathway.^95^ In clear cell renal cell carcinoma, AP000439.2, also delivered via exosomes, directly binds to STAT3 and activates the STAT3/NF‐κB pathway.^96^ They both promote tumor development by causing M2 polarization of TAMs. CircRNAs are “genomic dark matter” and play a crucial role in regulating gene expression and tumor behavior. However, its role in TAMs through TAEs has been less studied. But there is no doubt that they are of potential research value.

The ontogeny of exosomal cargo is the formation of various factors in the cells that secrete exosomes through assembly. Thus, in addition to various nucleic acid molecules, protein factors function as a regulatory substance in TAEs and act in the TME by means of intercellular communication. In HCC, intratumoral proteasome subunit alpha 5 (PSMA5) acts on TAMs via exosomes to activate the JAK2/STAT3 signaling pathway and promote M2 polarization.^97^ Similarly, in diffuse large B‐cell lymphoma (DLBCL), the subunit of the IL‐6 receptor, GP130, was significantly enriched in exosomes, activating STAT3 signaling and inducing M2 polarization in macrophages.^98^ NADPH oxidase 1 promotes malignant transformation of cervical cancer by inducing reactive oxygen species (ROS) and is enriched in exosomes secreted by tumor cells, delivered to TAMs likewise inducing ROS activation and promoting M2 polarization.^99^ The nucleotide‐binding oligomerization domain‐like receptor family pyrin domain‐containing 6 has a critical function in small cell lung cancer (SCLC) metastasis, where it acts on TAMs via SCLC‐secreted exosomes, inducing the activation of NF‐κB signaling and promoting M2 polarization.^100^

In general, the M1 polarization of TAMs exhibits a strong proinflammatory function and strong phagocytosis, and it promotes the transformation and reprogramming of TAMs that have been polarized to M2 type to M1 type through the secretion of a variety of oncogenic active factors. However, in some specific TMEs, TAMs exhibit a mixture of M1 and M2 types, with M1‐type TAMs playing a role in promoting cancer migration. Obviously, this type of phenomenon is not consistent with the consensus that M1‐type TAMs promote inflammation and suppress cancer.^101^ Moreover, such M1‐type TAMs are also regulated by TAEs. For example, in oral squamous cell carcinoma, thrombospondin 1 (THBS1) activates the p38, Akt, and SAPK/JNK signaling pathways of TAMs via TAEs and promotes M1 polarization of TAMs, but not M2 polarization.^102^ Previously, attention has been focused on the M2 polarization of TAMs. This study reveals that the function of TAMs after polarization is not absolute, and that there may be uncovered regulatory mechanisms behind it. there is a more complex link between the polarization of TAMs and tumor progression.

In summary, the TME is very closely linked to the malignant development of tumors, as a result of mutually regulated positive feedback. In the TME, the polarization of TAMs is the most important influencing factor affecting its regulatory function. M2 polarization of TAMs is regulated by various factors such as signaling pathways, is promoted during tumor progression through TAEs, and is involved in inducing behaviors such as tumor proliferation and migration. This is an important reflection of the interaction between the TME and tumor development. Although, little is known about the M1 polarization of TAMs, it is undeniable that M1‐type TAMs have a strong regulatory function, especially in some specific environments exhibiting cancer malignancy induction, which contradicts previous findings and deserves our attention.

### 3.3 Contribution of exosomes to drug resistance and immune evasion in cancer

With the advent of the postchemotherapy era, target and immunotherapies have made milestones in the treatment of cancer. For example, immune checkpoint inhibitors, such as partial programmed death‐1/programmed death‐ligand 1 (PD‐1/PD‐L1) inhibitors with cytotoxic T lymphocyte‐associated antigen‐4 (CTLA‐4) inhibitors have been approved for marketing by the United States Food and Drug Administration and the rest are in clinical studies.^103^, ^104^ However, it is inevitable that cancer counteracts tumor drug resistance by altering epigenetic and metabolic reprogramming, greatly limiting the clinical application of novel drugs.^105^, ^106^ Therefore, the search for targets to overcome antitumor drug resistance has now become an urgent challenge. Researches have shown that TAEs regulate cancer gene expression through cellular communication, inducing the development of tumor resistance and often accompanied by tumor proliferative and migratory behaviours.^45^, ^107^, ^108^ TAEs may be potential clinical therapeutic targets.

Under stress conditions, TAEs tend to contain components that induce tumor resistance to chemotherapeutic agents in order to modulate critical genes contributing to tumor resistance. In breast cancer (BC), chemotherapy‐induced secretion of miR‐378a‐3p and miR‐378d in exosomes is significantly upregulated and acts on adjacent BC cells, silencing Dickkopf 3 and NUMB and activating the stemness pathways of WNT and NOTCH, allowing BC to become chemoresistant.^109^ In addition, cancers are inherently resistant to chemotherapeutic agents, altering their gene expression through TAEs in the setting of chemotherapeutic agents in order to produce acquired resistance. Tumor exosome‐derived miR‐1260b serves as a tumor‐communicating substance, silencing homeodomain‐interacting protein kinase‐2 expression in human umbilical vein endothelial cells (HUVECs), and promotes the migration of tumor cells with the emergence of chemotherapeutic resistance.^110^ lncSBF2‐AS1 was significantly upregulated in glioblastoma (GBM) exosomes and translocated to adjacent GBM cells sponging miR‐151a‐3p, inducing the silencing deregulation of X‐ray repair cross complementing 4, and enhancing DNA double‐stand break repair, leading to chemoresistance.^111^ The conventional lysosomal pathway or the ubiquitin‐proteasome degradation pathway leads to the degradation of oncoproteins in chemosensitive cancer cells. The development of chemoresistance is often accompanied by dysregulation of protein degradation mechanisms, leading to the accumulation of oncoproteins within the cell. Moreover, the resistance mechanism is transmitted to chemo‐sensitive cells by means of cellular communication to transform them into drug‐resistant cells. For example, resistant TNBCs are induced to be resistant to gemcitabine by delivering annexin A6 (ANXA6)‐rich, which results in the inhibition of EGFR ubiquitination degradation in chemotherapy‐sensitive cells.^112^ Receptor–ligand interactions are not all direct, and TAEs act as an important mediator to mediate remote binding of receptors to ligands to amplify the impact of gene regulation. Resistant BC cells secrete exosomes enriched for the EPH receptor A2 (EphA2) protein, which translocates as a receptor to chemotherapy‐sensitive BC cells and binds Ephrin A1 ligand, thereby activating the downstream ERK1/2 signaling pathway and inducing chemotherapy resistance.^113^ This is not the classical receptor–ligand binding mode, but an exosome‐mediated reverse binding pathway. Previous studies have reported that tumor metabolic reprogramming is an important cause of chemotherapeutic resistance, and lipid metabolic reprogramming and ferroptosis inhibition are the metabolic pathway modulations that have received the most attention for inducing chemotherapeutic resistance.^114^, ^115^ TAEs are important regulatory mediators in the process of metabolic reprogramming of tumors. GC‐secreted exosomes are enriched for lncFERO and translocate to GC stem cells to interact with SCD1 mRNA, recruit heterogeneous nuclear RNP A1 (hnRNPA1) to promote its expression, and inhibit ferroptosis and chemoresistance.^116^ Previously, we described that TAEs promote tumor development by inducing the polarization of TAMs to interact with the TME. Indeed, M2 polarization of TAMs acts on tumor cells through a feedback mechanism and induces the development of chemoresistance. GBM exosomes are enriched for lnc‐TALC, translocate to the TME to promote M2 polarization in microglia and bind to Enolase 1 to promote MAPK phosphorylation and C5/C5a secretion, inhibit temozolomide‐mediated DNA damage, and induce chemoresistance.^117^

Furthermore, exosomes are important mediators of immune regulation in cancer as they provide a plethora of signals that can support or suppress immunosuppression of lymphoid and myeloid cell populations in tumors.^118^ Tumor‐derived exosomes promote the tumor immune response in two ways: on the one hand, by suppressing the function of dendritic cells (DCs), T cells, and NK cells. DCs, as mediators between innate and adaptive immune responses, play a crucial role in inducing the antitumor activity of T cells. DCs play an important role in the activation of the immune response. For example, exosomes produced by mouse BC cells target CD11b^+^ myeloid precursor cells in the bone marrow in vivo and inhibit their differentiation to DCs.^119^, ^120^ Lung cancer‐derived exosomes block DC differentiation by downregulating surface markers such as CD80, MHC‐II, and CD86 but upregulating the expression of CD11B and PD‐L1.^120^ In addition, exosomes can also inhibit T cell proliferation by converting extracellular ATP to adenosine. Adenosine inhibits a wide range of immune cells and stromal cells; therefore, higher levels of adenosine in tumors contribute to the immunosuppressive microenvironment. Tumor‐derived exosomes carry the exonucleotidases CD39 and CD73 on their surface, which convert ATP into extracellular adenosine, thereby inhibiting T cell proliferation.^121^ Study found that O‐GlcNAc transferase in the exosomes of esophageal cancer stem cells (ECSCs) was found to protect ECSCs from CD8^+^ T cell damage by upregulating PD‐1.^122^ NKG2D (NK cell‐activated receptor NK group 2, member D) is an activation receptor for NK, NKT, and CD8^+^ T cells, and its aberrant loss in cancer is a key mechanism of immune evasion, and NKG2D may be an exosome‐mediated immune evasion in cancer. NKG2D may be a physiological target for exosome‐mediated cancer immune evasion.^123^ The NKG2D receptor on NK cells is an important component of cancer immunosurveillance, as cancer cells typically express NKG2D ligands to varying degrees, signaling their response to NK cell‐mediated destruction.^124^ Such as, cancer cell‐derived exosomes expressing ligands for TGFβ and NKG2D downregulate the expression of NKG2D on the surface of NK cells and CD8^+^ T cells, thereby blocking its activity.^125^ On the other hand, tumor‐derived exosomes amplify the number of myeloid‐derived suppressor cells and regulatory T cells through various signaling pathways and stimulate macrophage polarization toward the procarcinogenic M2 phase.^126^ For example, tumor exosomes convert myeloid cells into MD⁃SCs by secreting PGE2, TGFβ or IL6, TNFα, and CCL2. TGFβ upregulates Foxp3+ T‐regulatory cell production in tumor exosomes.^120^ Finally, tumor‐derived exosomes may also advance cancer progression by developing xenogeneic satellite tumors alongside the primary tumor, which is called regional carcinogenesis, where morphological and molecular abnormalities occur around the tumor, although there are no obvious histological changes.

## 4 TAEs OF NUCLEIC ACID IN CANCER

With the rapid development of second‐generation sequencing technology, the research and clinical application of exosomal nucleic acids are also maturing. The sequencing of the human genome revealed that protein‐coding genes make up only 3% of human DNA, while more than 80% of the genome can be transcribed. These RNAs, which do not have the ability to code for proteins, are called ncRNAs.^127^

As research continues to evolve, our understanding of ncRNAs has shifted from “Junk” transcripts to functional regulatory molecules that mediate cellular processes such as chromatin remodeling, transcription, posttranscriptional modification and signal transduction.^18^, ^128^ Thousands of individual ncRNAs within the cell can form networks that influence numerous molecular targets to drive specific cell biological responses.^129^ As a key regulatory factor in physiological and disease processes, ncRNAs are closely related to tumors and have been identified as carcinogenic or tumor suppressor factors in a variety of cancer.^130^, ^131^ Many studies have shown that exosome ncRNAs have well stability due to the protective effect of exosome membranes. This stability holds great significance in the development of cancer.^132^, ^133^

In recent years, exosome‐derived miRNAs, lncRNAs, and other ncRNAs have attracted much attention in the field of cancer research worldwide. Thus, we summarize the roles of ncRNAs (with a focus on miRNA and lncRNA) in exosome ncRNAs in cancer. This summary aids to increase our understanding of the function of exosome ncRNAs in cancer and provide a basis for the development of antitumor therapeutic strategies based on exosome ncRNAs.

### 4.1 Exosmal miRNA in cancer

Precursor miRNA is a small RNA molecule with hairpin structure formed by transcription of RNA polymerase II and cleavage by Drosha. Subsequently, it is cleaved into a mature miRNA of approximately 23 nt in length, a process that involves Dicer. Eventually, the mature miRNA forms a complex with the corresponding RNA‐induced silencing complex, which exerts an essential gene silencing function mainly by binding to the 3′‐UTR of mRNA.^134^ Dysregulation of miRNAs promotes cancer development, and miRNAs are tightly associated with tumor metastasis in exosomes.^135^ Therefore, miRNAs carried by exosomes are very important gene regulatory signals between cells communication and are thought to have the potential to inhibit the metastatic properties of cancer.^136^ It was found that exosome secretion in lung cancer is regulated by signaling pathway such as LKB1 and AMPK/mTOR, which can promote cell motility.^137^ Besides, the investigators provided a comprehensive summary of the value of exosomal miRNA in the clinical diagnosis of lung cancer patients with EGFR mutations and the specific treatment regimen, including development of targeted drugs.^138^ In addition, there were more significant variations in the expression profiles of TAEs in metastatic cancer.^139^ These miRNAs differentially expressed in exosomes are likely to be underlying biomarkers of cancer metastasis. Plasma is widely present, abundant, and easy to detect. miRNAs in plasma exosomes of lung cancer patients are often used as clinical markers.^140^ Exosomal miRNAs derived from the plasma of lung cancer patients are often used as clinical diagnostic markers, including monitoring cancer metastases.^141^, ^142^ However, not all exosomal miRNAs have characterization value, which requires further validation.^143^, ^144^, ^145^

Exosomal‐delivered miRNAs may mediate the progression of drug resistance. For example, miRNA‐130a expressed by cancer‐associated fibroblasts derived from TME in cancer was packaged into exosomes by mutual effecting with the RNA‐binding protein PUM2. Eventually, miRNA‐130a promotes resistance to chemotherapeutic agents in lung cancer.^146^ In the case of EGFR mutations, clinically commonly treated with tyrosine kinase inhibitors are more likely to develop acquired resistance, which leads to metastasis of cancer.^147^ Previous studies speculated that the upregulation of miRNA in exosomes might be related to this. Kakeru Hisakane et al.^148^ identified exosomal miR‐210‐3p may promote acquired resistance to oxitinib in lung cancer cell lines and play a critical role in EMT. Due to the dense structure of tumors, the TME often reflects the characteristics of hypoxia. Exosome secretion under stressful conditions may have specific effects on cancer cells. Therefore, the hypoxic TME is closely associated with the occurrence and function of exosomes. High expression of miR‐625‐3p in exosomes of the hypoxic TME was internalized by lung cancer cells and binds to the 3′‐UTR of suppressor of cancer cell invasion and inhibited expression, eventually promoting lung cancer metastasis.^149^ Compared with the metastasis‐regulating mechanisms described above, the internalization of exosomal miRNAs by lung cancer cells revealed is not a direct effect on vascular epithelial cells. Therefore, exosomal miRNAs may have a more direct mechanism to promote metastasis in a hypoxic environment. EMT is a critical developmental process for tumor cells. Epithelial tumor cells regulate transcription factors and signaling pathway transduction through gene mutations and epigenetic modifications to enhance migration, invasion, and resistance to apoptotic stimuli and drug.^150^, ^151^ EMT confers local invasive ability to epithelial tumor cells and is therefore an important indication of tumor metastasis. Lung cancer cells with high metastasis play a signaling function to promote EMT in epithelial cells by secreting exosomes. With these exosomes, miRNAs play a crucial role in regulating gene expression. For example, miR‐224‐5p derived from lung cancer cells is internalized by the normal lung cell line BEAS‐2B via exosomal delivery and targets androgen receptor to promote invasion, migration, and EMT process.^152^ Derived from exosomes of highly metastatic lung cancer cells, miR‐499a‐5p enhances EMT in LUAD by activating the mTOR signaling pathway.^153^ A common process in lung cancer development involves lung cancer cells with high drug resistance promoting EMT in epithelial tumor cells through exosomal delivery of signaling molecules. The osimertinib‐resistant NSCLC cell line HCC827‐OR, along with PC‐9‐OR, promotes EMT in osimertinib‐sensitive lung cancer cells by secreting exosomes delivering miR‐210‐3p.^148^ In addition to this, it has been shown that some cells, such as MSCs, deliver message molecules via exosomes that perform the function of inhibiting EMT in lung cancer. MSCs deliver miR‐204 via exosomes and target Krüppel‐like factor 7 (KLF7) within the KLF7/AKT/HIF‐1α axis to inhibit EMT in cancer.^154^

As of present, more than a thousand endogenous exosomal miRNAs have been identified with potential biological functions. Therefore, the functions of exosomes are absolutely not homogeneous. For example, a study revealed that the exosomal delivery of miRNA‐494‐3p secreted by lung cancer cell into HUVECs and inhibition of PTEN expression exhibited antiangiogenic effects variously.^155^ In recent years, the therapeutic role of exosome miRNA in lung cancer has gained increasing attention,^156^ meanwhile the clinical treatment strategy of combining exosomal miRNAs with chemotherapeutic agents. Li et al.^157^ showed that exosomal miR‐613 secreted by lung cancer cells significantly enhanced the inhibitory effect of cisplatin on lung cancer and reversed the resistance of NSCLC to cisplatin. Moreover, the action of chemotherapeutic drugs may be closely associated with lung cancer exosome secretion.^157^ A study showed that everolimus targeting mTORC1 exerts therapeutic effects while inducing the secretion of miR‐7‐5p in the form of exosomes in NSCLC. Intracellular expression of miR‐7‐5p is reduced, leading to the release of inhibition of MAPK interacting kinases/eukaryotic translation initiation factor 4E (MNK/eIF4E) axis, as well as the resistance of everolimus.^158^ Therefore, induction of reinternalization of miR‐7‐5p‐containing exosomes in combination with everolimus is a potential clinical regimen for the treatment of NSCLC. In addition, engineering exosomes that introduce miRNAs with oncogenic effects may be a potential therapeutic strategy for future lung cancer clinics. Jeong et al.^159^ used 2D and 3D microfluidic techniques showed that engineered exosomes containing miRNA‐497 significantly suppress tumor growth‐associated genes in NSCLC. Besides, posttranscriptional silencing of VEGFR‐2 and inhibition of angiogenesis were achieved after miRNA‐497 was delivered into HUVECs via exosomes.^159^ This is an engineering exosome with potential clinical application.

Although exosomal miRNAs affecting cancer metastasis has been extensively explored for several years, more support and exploration are still needed toward the clinic. As mentioned before, in addition to developing new clinical diagnostic markers based on pivotal exosomes derived from cancer in regulating tumor metastasis, the combination of exosomal miRNAs with tumor metastasis therapy is also a direction worth exploring. How to further improve the targeting of engineered exosomes and combine them with traditional chemotherapeutic drugs to mitigate side effects is a hot topic of research now. miRNAs have received a lot of attention for cancer treatment, and there are already miRNAs moving toward clinical trials as drugs.^160^ It is clear that exosome delivery is an effective solution to improve the stability of miRNAs. In the future, intensive studies of exosomal miRNAs may provide more reliable clinical candidates for the early diagnosis and treatment of cancer progression (summarized in Table 1).

**TABLE 1:** Exosomal and miRNAs related with cancer progression by regulating cell function.

Cancer type/disease	Exosomes source	miRNA	Related genes or pathway	Functions	References
Lung cancer (NSCLC)	Lung cancer cells	miR‐934	circUSP7/SHP2	Promote resistance to anti‐PD1 immunotherapy	^161^
LUAD	Lung cancer cells	miR‐19b‐3p	STAT3	Promote lung adenocarcinoma metastasis	^92^
Lung cancer	Lung cancer cells	miR‐210	JAK2/STAT3 pathway	Proangiogenic factor	^47^
Lung cancer	Lung cancer cells	miR‐4466	STAT3	Promotes tumor cell stemness and metabolism	^162^
Colorectal cancer	Colorectal cancer cell	miR‐934	PI3K/AKT signaling	Induce M2 macrophage	^163^
Breast cancer	Breast cancer cells	miR‐205	E2F1	Promotes chemoresistance	^164^
Gastric cancer	Gastric cancer cells	miR‐501	BLID	Confers doxorubicin resistance and tumorigenesis	^165^
Lung cancer (NSCLC)	Serum	miR‐660‐5p	KLF9	Promote proliferation and migration	^49^
Lung cancer	Lung cancer cells	miR‐3180‐3p	FOXP4	Inhibits metastasis and proliferation	^166^
Lung cancer (NSCLC)	Paired tumor and normal tissue	miR‐4443	FSP1 m6A modification	Promote cisplatin resistance	^167^

Abbreviations: circUSP7/SHP2, circular ubiquitin‐specific protease‐7/Src homology phosphatase 2; E2F1, E2F transcription factor 1; FOXP4, Forkhead Box P4; FSP1, ferroptosis suppressor protein 1; KLF9, Krüppel‐like factors 9; STAT3, signal transducer and activator of transcription 3.

### 4.2 Exosomal lncRNA in cancer

A ncRNA molecule with more than 200 nucleotides is known as a lncRNA, which can regulate gene expression at the transcriptional or posttranscriptional level, thereby regulating various biological processes such as tumor cell growth, metastasis, and treatment resistance.^168^ Currently, numerous studies have demonstrated the connection between exosomal lncRNAs and the emergence of cancer.^169^, ^170^

Exosomes isolated from peripheral blood with NSCLC bone metastasis (BoM) were shown to be enriched in lncRNA–SOX2OT, according to research by Ni et al.^171^ and cancer patient's overall survival was inversely linked with the expression level of lncRNA‐SOX2OT. They further revealed that exosomal lncRNA‐SOX2OT derived from NSCLC cells targeted the miRNA‐194‐5p/RAC1 signaling axis, the TGF‐/pTHrP/RANKL signaling pathway in osteoblasts to regulate osteoclast development and trigger NSCLC BoM.^171^ Wu et al.^172^ demonstrated that TGF‐β1‐mediated exosomes increased lung vascular endothelial cell permeability and tight junction protein downregulation via lnc‐MMP2‐2. Additionally, lncRNA–MMP2‐2 upregulated EPR41L5 expression through sponge miR‐1207‐5p. EPR41L5 indirectly promoted EMT, disrupted tight junctions, increased blood–brain barrier (BBB) permeability and ultimately contributed to NSCLC brain metastasis.^172^ Studies also found that by targeting miR‐181a‐5p to control sirtuin 1 (SIRT1), Qu et al.^173^ showed that exosomal lncRNA DACT3‐AS1 deletion produced from CAFs increased proliferation, migration, and invasion of GC cells. Furthermore, through the combined actions of iron death and apoptosis, DACT3‐AS1 sensitized GC cells to oxaliplatin; iron death was more important for oxaliplatin sensitivity.^173^ Zhang et al.^174^ showed that the level of lncRNA MALAT‐1 in serum exosomes of NSCLC patients is significantly increased, which can promote the growth and migration of lung tumor cells and inhibit their apoptosis. Feng et al.^175^ identified exosome LINC00623 as a promising biomarker for the diagnosis of early pancreatic ductal adenocarcinoma (PDAC) with high specificity and sensitivity. The PDAC cells’ capacity for proliferation, tumorigenesis, migration, and invasion was supported by the LINC00623/NAT10 signaling axis. By modifying mRNA's N4‐acetylcytidine (ac4C) alterations, N‐acetyltransferase 10 (NAT10) improved oncogenic mRNA stability and translation in PDAC.^175^ Han et al.^176^, ^177^ screened the highly expressed lncRNA AFAP1‐AS1 from a trastuzumab‐resistant strain using lncRNA microarrays. In his research, he revealed that knockdown of AFAP1‐AS1 reversed trastuzumab resistance through gain‐of‐function and loss‐of‐function assays. Furthermore, extracellular AFAP1‐AS1 was able to bind to exosomes and deliver trastuzumab resistance. By interacting with AUF1, AFAP1‐AS1 increased the translation of ERBB2 mRNA. This increased the amount of HER‐2 protein, which led to trastuzumab resistance.^176^, ^177^ The expression of lncRNA lincRNA‐p21 was significantly increased in hypoxic NSCLC cells (H23 and HCC44), which promoted angiogenesis and endothelial adhesion of tumor cells.^178^ Cheng et al.^179^ studies have shown that the level of lncRNA GAS5 (growth arrest‐specific 5) is not only low in lung cancer tissues and serum exosomes, but also significantly lower in lung cancer cells (A549, H1299 and 95D) and their exosomes than that of normal bronchial epithelial cells. lncRNA GAS5 can competitively bind miR‐29‐3p with PTEN which upregulating the expression of PTEN and inhibiting the PI3K/protein kinase B (PKB/AKT) pathway. Additionally, the expression levels of HOTAIR and GAS5 are both affected by exosome secretion,^180^ and the downregulation of HOTAIR can increase NSCLC cell lines sensitivity to Crizotinib.^181^ Additionally, lung cancer‐derived exosomal GAS5 (exo‐GAS5) affects the apoptosis, proliferation, and tube formation of HUVECs.^179^

Drug resistance is a common clinical problem. Gefitinib and erlotinib belong to the generation of EGFR‐TKIs. Acquired drug resistance has become a major clinical problem with increasing use. Yu et al.^182^ concluded that exosomal lncRNA LOC85009 decreased docetaxel (DTX) resistance and cell growth but induced apoptosis. In addition, exosomes are used to transfer LOC85009 from LUAD cells to DTX‐resistant LUAD cells. Exosomal LOC85009 induced death in DTX‐resistant cells while inhibiting proliferation, autophagy, and DTX resistance. By controlling ATG5‐induced autophagy via the ubiquitin‐specific proteinase 5 (USP5)/upstream transcription factor 1 (USF1) axis, LOC85009 reduced DTX resistance.^182^ Through the development of a profeedback loop connected to the hnRNPA1/WNT5A/VEGFR3 regulatory axis, Zheng et al.^183^, ^185^ discovered a significant role for exosomal lncRNA BCYRN1 in bladder cancer's (BCa) lymphangiogenesis and lymph node (LN) metastasis. A new theory for how exosomal lncRNA induced tumor lymphatic metastasis was presented by the molecular mechanism by which exosomal BCYRN1 promoted LN metastasis of BCa in a VEGF‐C dependent way.^183^ According to Chen et al.,^184^ human lymphatic vessel endothelial cells internalized exosomal lncRNA LNMAT2 after uptake, and this increased PROX1 expression by enlisting hnRNPA2B1 and boosting the amount of H3K4 trimethylation on the PROX1 promoter, ultimately causing lymphangiogenesis and BCa lymphatic metastasis. Feng et al.^175^ identified exosome LINC00623 as a promising biomarker for the diagnosis of early PDAC with high specificity and sensitivity. The PDAC cells’ capacity for proliferation, tumorigenesis, migration, and invasion was supported by the LINC00623/NAT10 signaling axis. By modifying mRNA's ac4C alterations, NAT10 improved oncogenic mRNA stability and translation in PDAC.^175^ Furthermore, extracellular AFAP1‐AS1 was able to bind to exosomes and deliver trastuzumab resistance. By interacting with AUF1, AFAP1‐AS1 increased the translation of ERBB2 mRNA. This increased the amount of HER‐2 protein, which led to trastuzumab resistance.^176^ Wang et al.^186^ identified an adenomatous polyposis coli (APC)‐activated lncRNA (lncRNA‐APC1), which could increase the expression of lncRNA‐APC1 by preventing PPAR enrichment at its promoter. By directly binding to Rab5b mRNA and decreasing its stability, lncRNA‐APC1 prevented exosome formation, which in turn prevented CRC cell proliferation, metastasis, and tumor angiogenesis. The MAPK pathway was activated in endothelial cells by exosomes from CRC cells that have been repressed by lncRNA‐APC1, and this was significant because it promoted angiogenesis.^186^ Additionally, Lei et al.^187^ found that lncRNA H19 expression was significantly increased in gefitinib resistant cell line, and heterogeneous nuclear RNP A2/B1(hnRNPA2B1) can transfer gefitinib resistance by binding to the 5′ end‐specific motif (GGAG) of lncRNA H19 and mediating its loading into exosomes. Studies have also shown that patients with high expression of serum exosome lncRNA RP11‐838N2‐4 have erlotinib resistance. Notably, the high expression of RP11‐838N2‐4 can significantly increase expression in erlotinib‐resistant cells, and transferring of RP11‐838N2‐4 through exosomes can induce resistance insensitive cells.^188^

As the basic theories and applications of exosomal lncRNAs continue to be researched, exosomal lncRNAs will not only be used as biomarkers for early diagnosis, treatment monitoring, and prognosis assessment, but also as therapeutic targets for tumors, providing a novel approach to the accurate diagnosis and treatment of tumors.^189^ It can be seen that lncRNAs derived from tumor cells can regulate the biological behavior of cells through various mechanisms and affect the angiogenesis and disease progression of cancer. Therefore, exosomal lncRNAs will have a broad application prospect in the field of tumor diagnosis and treatment (summarized in Table 2).

**TABLE 2:** Exosomal and lncRNAs related with cancer progression by regulating cell function.

Cancer type/disease	Exosomes source	lncRNA	Related genes or pathway	Functions	References
Lung cancer (NSCLC)	Lung cancer cells	SOX2OT	miRNA‐194‐5p/RAC1	Regulate osteoclast development and trigger NSCLC BoM	^171^
Lung cancer (NSCLC)	Lung cancer cells	MMP2‐2	miRNA‐1207‐5p/EPB41L5	Promote NSCLC brain metastasis	^172^
Lung cancer (NSCLC)	Lung cancer cells	MALAT‐1		Promote growth and migration, inhibits apoptosis	^174^
LUAD	LUAD cell	LOC85009	USP5/USF1	Reduce docetaxel resistance	^182^
Pancreatic cancer	Pancreatic cancer cells	LINC00623	NAT10	Promote pancreatic cancer progression	^175^
Gastric cancer	Gastric cancer cells	DACT3‐AS1	miR‐181a‐5p/SIRT1	Promote ferroptosis‐mediated oxaliplatin resistance in GC	^173^
Bladder cancer	Bladder cancer cells	BCYRN1	VEGF‐C	Drive lymphatic metastasis of bladder cancer	^183^
Bladder cancer	Bladder cancer cells	LNMAT2	PROX1	Promote lymphatic metastasis in bladder cancer	^184^
Colorectal cancer	Colorectal cancer cells	APC1	Rab5b	Promote angiogenesis	^186^
Lung cancer (NSCLC)	Lung cancer cells	AFAP1‐AS1	AUF1	Promote trastuzumab resistance	^176^
Lung cancer	Lung cancer cells	lncRNA MMP2‐2	TGF‐β	Promote invasion and migration	^190^
HUVECs	Lung cancer	GAS5	PTEN	Promotes tumor angiogenesis	^179^
Lung cancer	Lung cancer cells	lncRNA H19	hnRNPA2B1	Promote gefitinib resistance by packaging into exosomes	^187^
Lung cancer	Lung cancer cells	lncRNA RP11‐838N2. 4	FOXO1	Promote erlotinib‐resistant	^188^

### 4.3 Other ncRNAs (circRNA/tRF) from exosome in cancer

Among exosomal, cancer is also closely associated with a number of other ncRNAs (circRNAs and tRF), except miRNAs and lncRNAs. circRNA is a new member of endogenous ncRNA, which is widely distributed and has diverse cell functions. Recently, circRNAs have been discovered due to their stability and enrichment in exosomes.^191^ Studies have found that some circRNAs are associated with tumor development, progression, and drug resistance. Exosome and circRNA_101093 (cir93) are significant for insensitivity LUAD cells to ferroptosis in which blocking exosome may help with LUAD treatment.^192^ Peng et al.^193^ identified circRNA CD226 relies on regulation of the miR‐1224‐3p/HMGA2 axis as a potential driver of cancer development. Tumor‐derived exosomal circRNAs have been reported to be key circRNA in the process of EGFR‐TKIs resistance. Tumor‐derived exosomal circRNA_102481 may promote EGFR‐TKIs resistance in NSCLC via the miRNA‐30a‐5p/ROR1 axis.^194^ Additionally, knockdown of exosomal circ_PIP5K1A could suppressed NSCLC development and promoted cisplatin sensitivity via regulating miR‐101/ABCC1 axis.^195^ In LUAD patients, both the serum and serum exosomal circRNAs, hsa_circ_0001439, hsa_circ_0001492, and hsa_circ_0000896 were upregulated. Serum exosomal circRNAs may be a more effective biomarkers than serum circRNAs in the diagnosis of LUAD and may further benefit the detection of this disease.^196^ Thus, exosomal circRNAs have potential applications as new therapeutic targets and disease biomarkers, although their specific roles and gene regulatory mechanisms require more investigation.

tRFs are also small RNAs that come from the 5′ end or 3′ end of the tRNA in a specific environment.^197^, ^198^ The biological function of the derived exosome tRFs is still ambiguous. Studies have found that the levels of exosomes tRFs (tRF‐Leu‐TAA‐005, tRF‐Asn‐GTT‐010, tRF‐Lys‐CTT‐049, tRF‐Ala‐AGC‐036, and tRF‐Trp‐CCA‐057) in patients with cancer are downregulated.^199^ This suggests that these exosomal tRFs may be diagnostic biomarkers for cancer. Wang et al.^200^ found that alveolar macrophage‐derived exosome tRF‐22‐8BWS7K092 can activate Hippo signaling pathway to induce iron apoptosis in acute lung injury, BALF exosomes in ALI mice are mainly derived from AMs. Currently, little is known about exosomal and TRF expression in cancer. Nonetheless, their biological functions have attracted the attention of many studies. In the future, exosomal tRFs may act as latent diagnostic biomarkers and therapy target in cancer.

## 5 THERAPEUTIC TARGETING OF TAEs

Exosomes have been shown to play a role in cancer progression and metastasis by promoting the exchange of genetic material and proteins between cancer cells and their microenvironment. However, exosomes can also be harnessed for therapeutic purposes, including the treatment of cancer. The multiple components and properties that exosomes can carry make them potential therapeutic targets in cancer therapy. Through in‐depth study of the mechanism of exosomes' role in cancer genesis, progression and drug resistance, and the development of therapeutic approaches against these targets, it is expected to bring new breakthroughs in cancer therapy.

### 5.1 Strategies for inhibiting exosome secretion and uptake in cancer cells

One potential application of exosome‐based therapies in cancer patients is the delivery of therapeutic agents directly to cancer cells. Exosomes can be engineered to carry drugs, small interfering RNA (siRNA), or other therapeutic molecules and can be targeted to specific cancer cells.^201^, ^202^ Such as exogenous siRNA to monocytes and lymphocytes in human blood cells, which exosomes can silence the MAPK1 gene.^203^, ^204^ This demonstrates that exosomes can deliver therapeutic RNA to target cells. This approach has the potential to increase the efficacy and specificity of cancer therapies while minimizing side effects. Another potential application of exosome‐based therapies is in the field of immunotherapy.^205^ Tumor immunity usually refers to the activation of the immune system when immune cells infiltrate and come into contact with the tumor, and to the ongoing death of tumor cells to free up antigen. This is followed by the activation of T and NK cells and the production of antibodies to destroy the tumor cells.^206^, ^207^ Yang et al.^208^ found that in glioma, tumor‐derived exosomes inhibit CD8^+^ T‐cell proliferation, immune cell activity, and IFN‐γ levels by inhibiting PEG3, thereby inhibiting glioma cell apoptosis and further enhancing glioma cell immune evasion through in vitro and in vivo experiments. M2 macrophages which secreted from exosomes significantly enhanced the proliferation and antiapoptotic ability of colon cancer. Lu et al.^209^ found that FOXO3‐AKT/GSK3β pathway and the mechanism by which TAM‐derived exosomal miR‐29a‐3p upregulates PD‐L1 expression to facilitate OV cell proliferation and immune escape. This study is through inhibiting T‐cell activity and ultimately facilitating immune evasion. Moreover, the researchers showed that M2 macrophage‐derived exosomes can promote GC progression by activating the P38MAPK pathway, while also promoting high PD‐L1 expression in GC cells, which aids GC cells in immune evasion and leads to poor prognosis.^210^ Therefore, to overcome the immune evasion of tumors, targeting exosomes derived from tumor may be an effective strategy.

Exosomes can be engineered to carry immune‐stimulatory molecules that can activate the immune system and promote an antitumor response which has shown promise in preclinical studies.^211^ There are several challenges that need to be overcome to fully realize the potential of exosome‐based therapies in cancer patients. These include the optimization of exosome production and engineering, the identification of suitable cancer targets, and the development of strategies to overcome drug resistance and tumor heterogeneity.

### 5.2 Potential therapeutic targets within exosome cargo for cancer treatment

**FIGURE 2:**
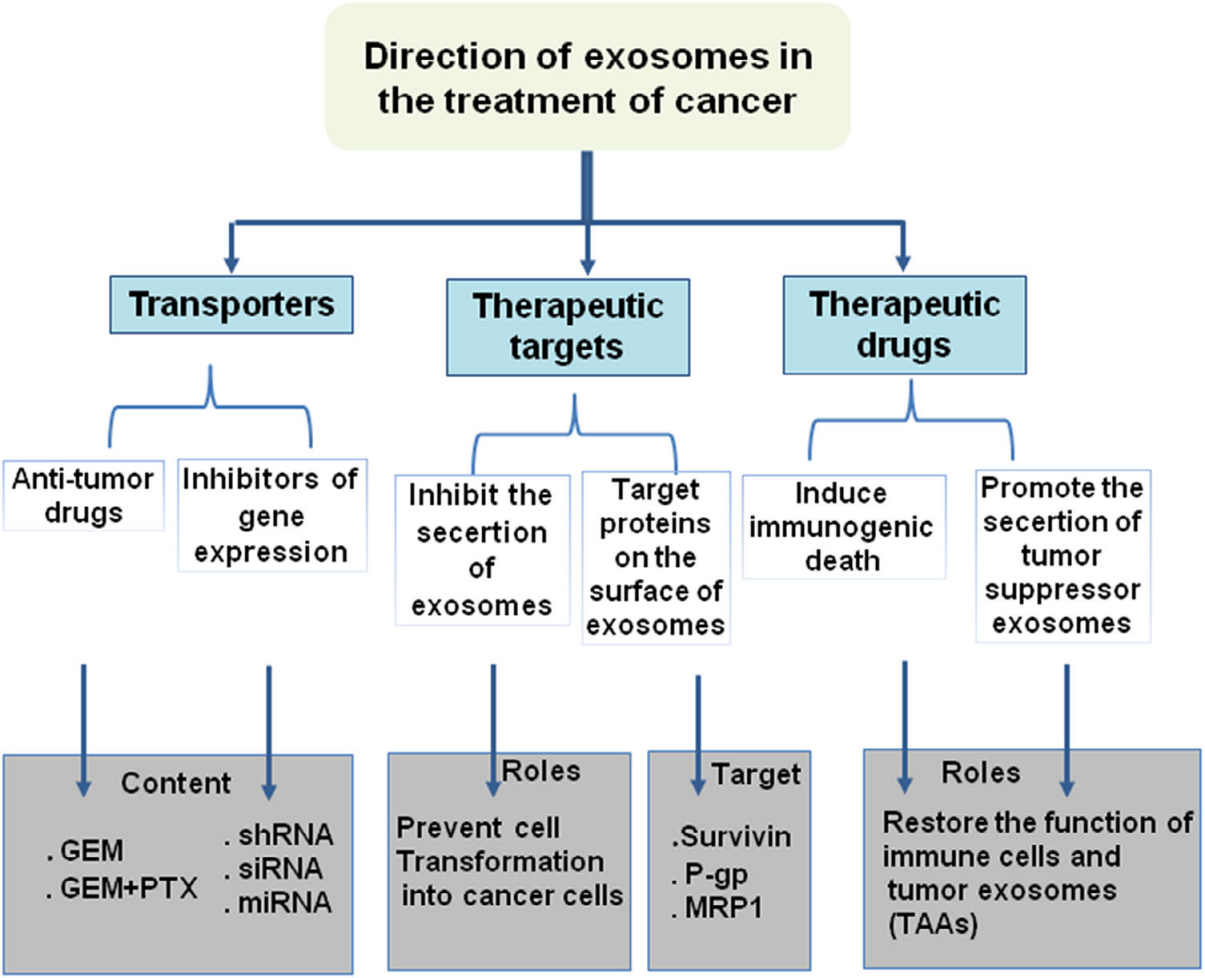
Directions in exosome therapy for cancer. GEM, gemcitabine; GEM + PTX, gemcitabine + paclitaxel; P‐gp, P‐glycoprotein; MRP1, multidrug resistance‐associated protein 1; TAAs, tumor‐associated antigens.

Exosomes are secreted by a variety of cells, including cancer cells, and carry a variety of molecules, including proteins, lipids, and nucleic acids.^188^, ^212^ These molecules, collectively referred to as the exosome “cargo,” can have significant effects on the TME and contribute to cancer progression and metastasis. Several potential therapeutic targets within exosome cargo have been identified for cancer treatment: (1) *Oncogenic proteins*: Exosomes can carry oncogenic proteins that promote cancer cell growth and survival.^213^ Inhibiting the secretion or function of these proteins could be a potential therapeutic strategy. (2) *miRNAs*: Exosomes can carry miRNAs that regulate gene expression and promote cancer progression. Such, by secreting ACSL4‐targeting miRNAs produced from exosomes, CAFs inhibit ferroptosis and cause PC cells to become resistant to gemcitabine.^214^ Inhibiting the secretion or function of these miRNAs could be a potential therapeutic strategy. (3) *Tumor‐associated antigens (TAAs)*: Exosomes can carry TAAs, which are proteins that are overexpressed in cancer cells and can be recognized by the immune system.^215^ Targeting TAAs with immune‐based therapies could be a potential therapeutic strategy.^216^ (4) *Immune checkpoint proteins*: Exosomes can carry immune checkpoint proteins, such as programmed cell death protein 1 (PD‐1) and cytotoxic T‐lymphocyte‐associated protein 4 (CTLA‐4), which can inhibit immune responses against cancer.^217^ Blocking these proteins could be a potential therapeutic strategy. (5) *Drug resistance proteins*: Exosomes can carry proteins that contribute to drug resistance, such as P‐glycoprotein (P‐gp) and multidrug resistance‐associated protein 1.^218^ Inhibiting the secretion or function of these proteins could be a potential therapeutic strategy. (6) *Tissue factor (TF)*: Exosomes can carry TF, a protein that plays a role in blood clotting and can promote cancer metastasis.^219^ Inhibiting TF function could be a potential therapeutic strategy. In summary, in combination with the characteristics of exosomes, their application in the treatment of cancer has mainly the following directions (Figure 2), the exosome cargo contains several potential therapeutic targets for cancer treatment.

Qiu et al.^39^ found that GC exosomal miR‐519a‐3p promotes liver metastasis through induction of intrahepatic M2‐like macrophage‐mediated angiogenesis. This study provides experimental evidence supporting the potential therapeutic targeting of miR‐519a‐3p in GC. Moreover, researchers elucidated that tumor‐derived exosomal miR‐148b‐3p can be transported from BC cells to surrounding macrophages and induces M2 polarization by targeting TSC2, providing new insights for BC therapy.^220^ These findings lay the groundwork for future research on BC potential therapy. Emerging evidence suggests that exosomes may act as cargo, facilitating intercellular communication and allowing tumor cells to signal surrounding stromal cells to alter cancer progression.^221^, ^222^ Even so, further research is needed to fully understand the role of these targets in cancer progression and to develop effective therapies that target them.

### 5.3 Clinical applications of exosome‐based therapies in cancer patients

At present, exosomes show great potential in clinical treatment of cancer because of their ability to carry and deliver important substances, including therapeutic molecules. Compared with traditional drug delivery systems, exosomes are biocompatible, low immunogenicity, and highly targeted. They are able to penetrate biological barriers, such as the BBB and deliver therapeutic molecules directly to the lesion site.^223^, ^224^ One of the important application directions is that exosomes can be used as biomarkers for cancer patients. Wei et al.^225^ found that showed that the combination of exosome‐EphA2 + CA19‐9 + CA242 can not only improve the ability to diagnose PC at an early stage, but also improve the ability to differentiate PC from benign pancreatic diseases, using exosome‐EphA2 + CA19‐9 + carbohydrate antigen 242 (CA242) as an early diagnostic marker. Li et al.^226^ demonstrated that serum exosome levels were significantly elevated in patients with OV compared with benign disease or healthy controls, and that 117 differentially expressed lncRNAs as well as 513 differentially expressed mRNAs were dysregulated in expression as revealed by mapping between mRNAs of exosome origin and those of corresponding tumor origin. Therefore, tailoring cancer treatment by analyzing the various ribonucleic acids contained in exosomes for risk assessment of patients may be achieved in the near future.

Additionally, exosomes as new drug carriers also have many advantages. Clinical trials on exosome therapies have already begun, such as the exosome group transporting a small molecule STING agonist, a phase I/II clinical trial for advanced solid tumors.^227^ Jonathan et al.^228^ reported that exosomes from the engineered melanoma cell contain survivin‐T34A and, alone or in combination with gemcitabine, significantly increased apoptotic cell death compared with gemcitabine alone in a variety of PC cell lines. Their research shows that novel exosome‐mediated delivery of survivin T34A mutant increases gemcitabine sensitivity in pancreatic adenocarcinoma. Current experimental stage, the methods commonly used in recent years include chemical linking of targeting peptides, modification of exosomal membranes or progenitor cells by genetic engineering, magnetic nanoparticle technology, electrostatic interaction, and postinsertion. These methods are all aimed for precisely target the therapeutic agent to the lesion. Applications of partial surface modification exosomes are summarized in Table 3.

**TABLE 3:** Clinical applications examples of exosome‐based therapies in cancers.

Target mode	Targeting method	Drug loading	Targeted tissues and applications	References
Chemical reaction	Extracellular vesicles containing azide lipids were conjugated to the targeted peptide using copper‐free catalytic click chemistry.	PTX (loaded on liposomes)	Tumor cells	^229^
Genetic engineering	Engineered mouse immature dendritic cells expressing with Lamp2b fused to iRGD peptide to generate tumor‐targeted exosomes.	DOX (electroporation)	Tumor tissues, suppress the growth of tumor	^230^
Genetic engineering	In dendritic cells (dc), expression of the membrane protein‐containing fusion proteins Lamp2b and RVG peptides, as well as exosomes, are available from the cells by genetic modification.	siRNA (electroporation)	Targeting the central nervous system (neurons, microglia, oligodendrocytes) to treat Alzheimer's disease	^231^
Genetic engineering	Donor cells were engineered to express platelet‐derived growth by targeting transmembrane domain factor receptors fused with GE11 peptides in order to absorb exosome sources from them.	let‐7a miRNA (transfection)	Breast cancer tissue targeted to express EGFR for breast cancer treatment	^232^
Electrostatic interaction	Complexes formed by cationic lipids and sensitive fusion peptides bind exosomes through electrostatic interactions to target the receptor cell membranes.	Dextran, saponin (electroporation)	Targeting receptor cell membranes enhances cellular uptake and cytoplasmic release of exosomes	^233^

Abbreviations: DOX, doxorubicin; PTX, paclitaxel; siRNA, short interfering RNA.

There are several challenges that need to be overcome to fully realize the potential of exosome‐based therapies in cancer patients. These include the optimization of exosome production and engineering, the identification of suitable cancer targets, and the development of strategies to overcome drug resistance and tumor heterogeneity.^45^ Furthermore, targeting TAEs may also have diagnostic potential, as their cargo can reflect the molecular profile of the originating tumor.^234^ This could allow for noninvasive monitoring of treatment response and disease progression. Overall, therapeutic targeting of TAEs holds great promise as a novel approach to cancer treatment, offering the potential for more effective and personalized therapies. With the continuous progress of science and technology and the deepening of clinical trials, we believe that exosomes will become one of the important tools for cancer treatment in the future.

## 6 CONCLUSIONS

Exosomes are vital carriers of information transmission between tumor cells, which affect the proliferation, metastasis, and invasion of cancer via regulating the microenvironment of tumor.^235^ Research mechanism of exosomal in cancer has achieved notable breakthrough, such as the clinical application of antivascular and EGFR‐TKIs resistance mechanism.^236^ Exosomes also play a crucial role in tumors combined with other diseases, such as some neuropsychiatric diseases^237^ and ocular diseases.^238^ It is believed that exosomes may be more widely applied in the clinical diagnosis, prognosis prediction and treatment of cancer in the near future.

**FIGURE 3:**
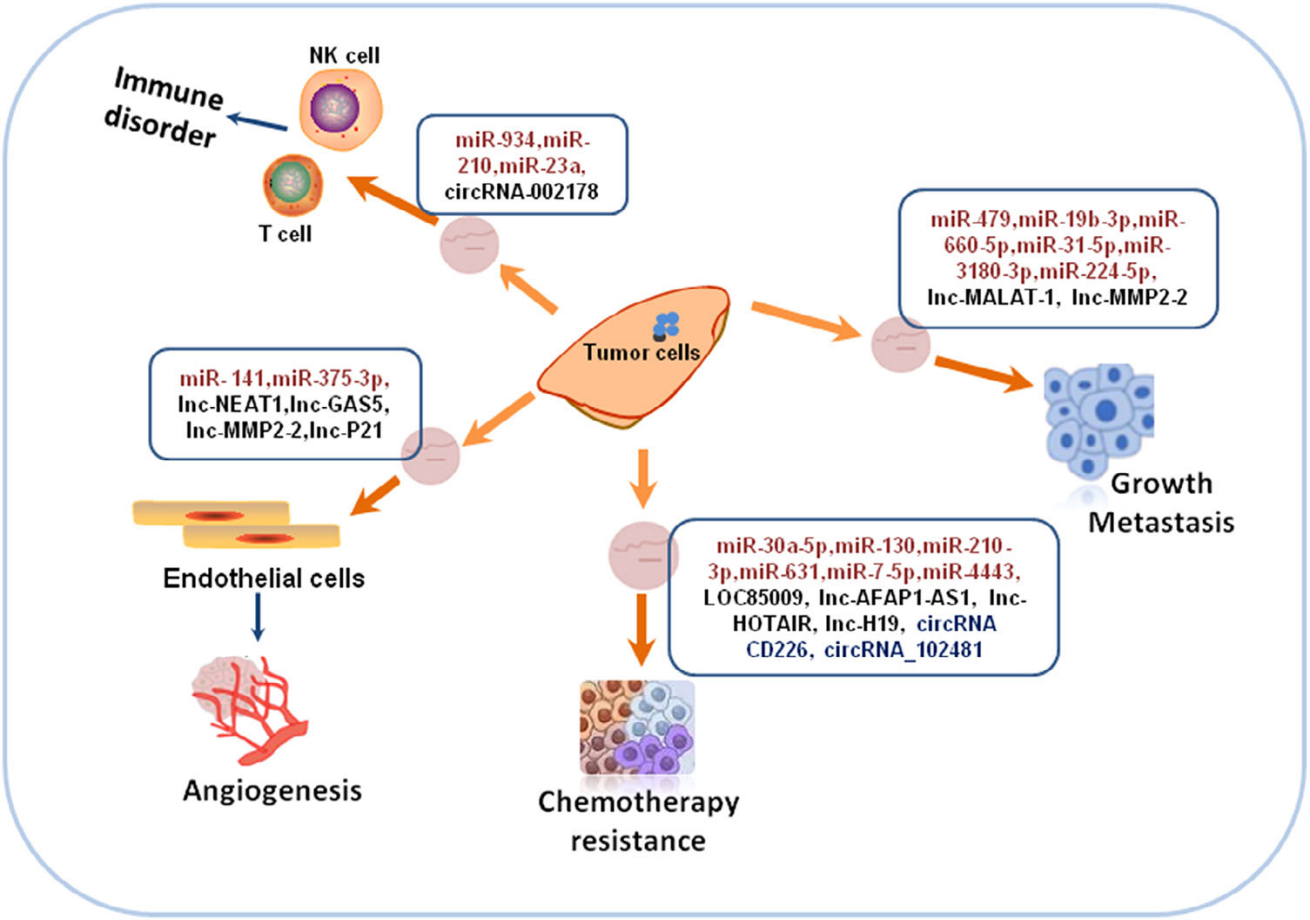
The function of exosome noncoding RNAs in cancer. Tumor cells secrete exosomes, which contain diverse nucleic acids (ncRNA) to modify tumors cells and stromal cells in the tumor progression such as endothelial cells to effect angiogenesis, tumor cells develop resistance to drugs, growth metastasis, and immune disorder.

Exosomes ncRNAs (miRNAs, lncRNAs, and other ncRNAs) act as an important role in cancer tumor progression (Figure 3). Cancer cells can secrete exosomes containing specific ncRNAs to other cancer cells, endothelial cells, mesenchymal cells, and immune cells, activate receptor cell signaling pathways, regulate the expression of target genes, induce angiogenesis, EMT, and immune escape, and so on, and promote the occurrence and development of cancer.^239^, ^240^ On the contrary, immune cells, mesenchymal cells, endothelial cells, and other stromal cells can also transmit exosomes to cancer cells, affecting the proliferation, invasion, and metastasis of tumor cells.^45^, ^241^ However, many people with cancer are diagnosed at an advanced stage. It is hoped that more comprehensive research on the pathogenesis of tumors by exosomes can make a breakthrough in the early diagnosis of tumors, and bring more benefits for the early detection, early diagnosis and early treatment of patients.

In general, exosomes are important pathways for tumor cell information transmission. Nowadays, the study of exosomes in the mechanism of cancer has achieved many positive results. However, there is a lack of in‐depth systematic research at present. There are still many questions about tumor‐derived exosomes and mechanism of cancer metastasis. For instance, how tumor‐derived exosomes and cancer cells or host cells identify each other and how vesicle contents play a role in regulating tumor progression. Are there stronger genes signal in tumors than that derived from exosomes or expressed by metastatic tumor cells? Thus, there needs to be more researches on both the basic and clinical translational studies on exosomes and tumor. Given the role of exosomes, future researches will afford better guidance for the future diagnosis and treatment of cancer, especially the diagnosis and clinical treatment of progression of cancer.

## AUTHOR CONTRIBUTIONS

*Investigation and writing—original draft preparation*: Xiaomin Liu, Fan Wu, Wei Pan, Guangchao Liu, Hui Zhang and Dawei Yan. *Investigation and writing—review and editing*: Xiaomin Liu, Fan Wu, Wei Pan, and Xiaojun Ren *Conceptualization, investigation, writing—original draft preparation, supervision, and project administration*: Zhongliang Ma, Xiaomin Liu, Fan Wu, Hui Zhang, Saijing Zheng and Xiaojun Ren All authors have read and agreed to the published version of the manuscript.

## CONFLICT OF INTEREST STATEMENT

Authors X. M. L., G. C. L., H. Z., D. W. Y., and S. J. Z. are employees in Shanghai New Tobacco Product Research Institute Co., Ltd. The other authors have no conflict of interest to declare.

## ETHICS STATEMENT

Ethics approval was not needed for this study.

## Data Availability

Not applicable.
